# Is There a Continued Fever Other Than Typhoid?

**Published:** 1906-06

**Authors:** J. W. Palmer

**Affiliations:** Ailey, Ga.


### IS THERE A CONTINUED FEVER OTHER THAN MALARIAL OR TYPHOID?*

BY J. W. PALMER, M.D.,

AILEY, GA.

So much has been said and written on this subject, no doubt a great many of you feel that it is threadbare; but the fact is, the last word has not been said, nor will be for some time.

I have nothing new to add to the subject, and will not speak of it from a pathological or etiological standpoint, but from a bedside study.

The Georgia State Board of Health having decided to locate a station in the southern part of the State to investigate and see if they could discover what this third fever was, and I having had an unusual amount of continued fever in my practice for the past

* Read at Augusta before Georgia Medical Association, April 18, 1906.

eight months, I felt that I might revoke a discussion of the subject if nothing more.

What is fever? We mean by fever an abnormal elevation of the temperature of the body with more or less pronounced functional derangement of all the organs of the body, lasting for some time, or repeated at certain intervals, and not produced by external heat or violent muscular exercise. This abnormal elevation of temperature is supposed to be caused either by direct action of micro-organisms upon the nerve centers to which they are carried by the blood, or by the action of a poison which they develop in the body. Wood claims that the inhibitory center is so depressed and benumbed by these organisms that it does not exert its normal influence upon the system, and consequently tissue change goes on at a rate which results in more heat than normal; at the same time the vasomotor and other heat dissipation centers are so benumbed by them that they are not called into action by their normal stimulus. Fever is classified, as continued, remittent and intermittent, according to the character of the temperature range in either case.

Now, in regard to there being a continued fever other than typhoid or malarial, I don't believe there is. My knowledge of the subject, based on my experience with continued fever, and from what I have read on the subject, forces me to represent the negative side of this question.

Since last June I have tabulated forty-eight cases of continued fever, of which thirty-six were typhoid, and the remainder were malarial. I kept close tab with all these cases, and not in a single instance did I not satisfy myself as to the fact that it was typhoid or malarial, and not a new fever.

From a clinical standpoint, I believe all these questioned cases of continued fever are a variety of mild, uncomplicated cases of typhoid fever, except occasionally it is malarial. I live near malarial districts, and I have taken notice that this continued fever seldom occurs much in localities where remittent and intermittent fever prevail. In my tabulated typhoid cases there were a few families through whom the fever went, and scarcely any of the members escaped. Some members of the family had typical cases of typhoid, while the majority had mild atypical cases, affording me a good opportunity to study these atypical cases of typhoid, which I could exclude from malarial or some unknown continued fever.

I will mention a few of these cases of atypical typhoid that occurred in families where some of the members of the family preceded them with malignant cases of typhoid.

*Case No. 1.*—Boy, twelve years old, white; had a continued fever for twenty-three days, ranging from 100 to 103½ degrees, with no symptoms whatever of typhoid fever, except the rose-colored spots on the abdomen. Had I seen this boy first in the family and those rose-colored spots absent, I would have been inclined to call it malarial, or this unknown fever.

*Case No. 2.*—White, female, age fifty; run temperature from 100 to 102½ degrees for only fourteen days, but seemed very sick during this period, and especially suffering from severe pain in hepatic region; had no symptoms of typhoid, more than continued fever. Quinine had no effect and malarial plasmodium in the blood was found wanting. Had I seen this case elsewhere, I would not have called it typhoid fever.

*Case No. 3.*—Boy, colored, age sixteen, came to my office apparently very sick to be out; had temperature 101½ degrees, feeble pulse, and a severe bronchial cough. I could not tell at that time whether he had consumption, malarial or typhoid fever, but giving a history of being sick only ten days, and having a sister who had been down for two months with fever, I placed him on typhoid treatment. This boy never went to bed, but in ten more days was able to do light work. I later treated four more members of the family for typhoid fever. Had it not been for the typhoid history in the family, I would never have known for certain that this boy had typhoid fever. I could report several more cases similar to these, but will report a few cases that occurred sporadically that were proved typhoid by complications.

*Case No. 4.*—Woman, white, age twenty-three years; had two children. Had smallpox eighteen months prior; had convulsions at birth of last child, ten months ago; also had psoriasis. When I saw her she had continued fever, with no symptoms of typhoid whatever, temperature ranging from 100 to 102 degrees. Her symptoms were so mild I could not keep her in bed. On the twentieth day she began to have intestinal hemorrhages and continued until the twenty-third day, when she died.

*Case No. 5.*—Man, colored, age twenty-five years; came to my office with continued fever and other symptoms of malarial. Other members of the family had done likewise, and with malarial treatment fever had yielded in few days. I placed this case on similar treatment, and told him to go to bed and if not better in few days to send for me. I heard nothing more until eight days later he returned to my office, saying he was better, but not well. I then saw he had something more than malarial, and told him to stay in bed, and I saw him every day for a week, at the end of which, during my absence, he had a fatal intestinal hemorrhage. This case never showed any symptoms of typhoid, only by exclusion, as quinine had no effect, and I could not find any malarial placomodium in the blood.

*Case No. 6.*—Woman, white, married, age twenty-two; one child; had a combined fever for twenty-eight days with temperature ranging from 101 to 103 degrees with no symptoms of typhoid. I was treating this case for autumnal malarial, as it is claimed that quinine has no effect on this form of malarial and it is usually very difficult to find the malaria plasmodium in the blood. After the twenty-eighth day this case began to have repeated profuse intestinal hemorrhages for three days, after which she made an uneventful recovery.

I could report other similar cases, but this suffices to show that had it not been for the previous typhoid history in the first cases, and the typhoid complications in the last cases, I would never have felt fully satisfied as to the kind of fever I was dealing with, but would have been inclined to believe it malarial or this unknown third fever in question. Hence, this leads me to believe that all these cases thought to be a third fever when brought down to a correct diagnosis, will be proven typhoid. These last cases I reported should also impress us with the importance of having all suspected cases of typhoid to go to bed at once, because I believe had these cases stayed in bed from beginning of fever, they would have now been living.

When this continued fever, long ago, was called typho-malarial, it was thought they then had a third fever, and even now when we are doubtful about our diagnosis as a catch-all, we call it typho-malarial; so, whichever one it turns out to be, we will say that element predominated and make ourselves safe. There is no such fever as combination of typhoid and malarial. Prof. Osler claims that the only way to have both of these fevers at the same time is while suffering with malarial, you may at the same time have the typhoid bacilli in your system, which may begin to develop typhoid, while you still have some malarial; that they are two distinct germs, one bearing no relation to the other whatever, and there is no such thing as these germs uniting and producing a third fever known as typho-malarial. There is no such disease as typho-malarial fever, because the cases called typho-malarial are nothing but simple uncomplicated cases of typhoid fever of a mild form; they are only a clinical variety of typhoid fever because it must be remembered that uncomplicated typhoid often presents a decidedly remittent character at the onset of the attack. The non-recognition of this fact leads to frequent mistakes in diagnosis and that many cases of simple typhoid, and especially its mild cases, are imperfectly classified under the headings, malarial, typho-malarial and remittent fever. In these cases we usually make our diagnosis at an early period in the progress of the disease, before the distinctive characters of typhoid fever have been developed, and at a time when the fever is very often remittent in its character.

We find in the first medical volume of the Medical and Surgical History of the War of Rebellion, that continued fever was changed to typho-malarial, and later the board of medical officers recommended change in nomenclature from typho-malarial to typhoid.

Prof. Osler, in one of his New York addresses, in speaking of this third fever, where, in New York, they called it malarial to keep from speaking of it as typhoid fever, he called it New York malarial, which is one of his synonyms of typhoid.

Dr. Wood, in his "Practice of Medicine" (ninth edition), says: "In some instances typhoid fever presents no other symptoms than those of moderate fever. The tongue remains soft, moist and whitish throughout; there is no vomiting, no considerable nervous disorder, no great prostration; in fine, none of these peculiar symptoms commonly dominated typhoid. The disease runs its course in two or three weeks, sometimes in even less time, and then subsides spontaneously, leaving no unpleasant effects."

This fever occurs mostly in autumn and winter months, and can be traced usually to insanitary conditions. All the fatal cases of this class where postmortems were held were found to be typhoid fever. Where remittent and intermittent fever are prevalent, you don't find much of this type of fever, hence this all goes to prove there is no third fever.

In handling these cases of continued fever, we should be very careful to make a correct diagnosis before we entertain the idea of a new fever. We must use all means and methods at our disposal to exclude malarial and typhoid, and especially typhoid. To exclude malarial, first cinchonize our patient; second, make microscopical examinations of the blood in search of the malarial plasmodium, and if results negative you may feel safe in excluding malarial unless it might be the autumnal malarial, and you may further exclude by trying the typhoid test, which, if affirmed, certainly exclude malarial. As soon as we have excluded malarial and doubt typhoid fever, we should at once send Dr. Harris, of Atlanta, a specimen of blood, requesting him to wire us the results. The best means of examining the blood at home is with an apparatus gotten out by Parke,

Davis & Co., called agglutometer. I consider it a very safe and reliable test, and I recommend it to all those who have not tried it. There are full directions with it, which are too lengthy to mention here.

I think that is one of the wisest movements of the State Health Board, when they suggested a laboratory stationed in Southwest Georgia to investigate this continued fever. It is the duty of the doctors of the State to urge its establishment at once. I believe it will help to settle this question of a third fever and cause more of us to make a correct diagnosis of typhoid fever.

In short, I say when quinine will have no effect on this fever, and you can't find any malarial "bugs" in the blood, it should be treated as a case of typhoid fever, and we should continue to do this until those who maintain the existence of a distinct form of continued fever, discover its cause, define its character and give it a specific name.

If, as some may claim, that there is a form of continued fever widely prevalent, which is influenced by the same predisposing causes, but is distinct from the typhoid or malarial fever, the burden of proof is upon them, and we should never except it until proven.

